# Challenges to Online Medical Education During the COVID-19 Pandemic

**DOI:** 10.7759/cureus.8966

**Published:** 2020-07-02

**Authors:** Mohammad H Rajab, Abdalla M Gazal, Khaled Alkattan

**Affiliations:** 1 Epidemiology and Public Health, Alfaisal University, Riyadh, SAU; 2 Internal Medicine, Alfaisal University, Riyadh, SAU

**Keywords:** coronavirus pandemic, covid-19, alfaisal university, online medical education, face-to-face-instruction

## Abstract

The coronavirus disease 2019 (COVID-19) pandemic has impacted all aspects of our lives, including education and the economy, as we know it. Governments have issued stay-at-home directives, and as a result, colleges and universities have been shut down across the world. Hence, online classes have become a key component in the continuity of education. The present study aimed to analyze the impact of the COVID-19 pandemic on online education at the College of Medicine (COM) of Alfaisal University in Riyadh, Saudi Arabia. Between March and April 2020, we emailed a survey to 1,289 students and faculty members of the COM. We obtained 208 responses (16.1%); 54.8% of the respondents were females, and 66.8% were medical students; 14.9% were master’s students, and 18.3% were faculty. Among the respondents, 41.8% reported having little or no online teaching/learning experience before the pandemic, and 62.5% preferred blending online and face-to-face instruction. The reported challenges to online medical education during the COVID-19 pandemic included issues related to communication (59%), student assessment (57.5%), use of technology tools (56.5%), online experience (55%), pandemic-related anxiety or stress (48%), time management (35%), and technophobia (17%). Despite these challenges, most of the respondents (70.7%) believed that the COVID-19 pandemic has boosted their confidence in the effectiveness of online medical education. Consequently, 76% of participants intended to integrate the online expertise garnered during the pandemic into their practice. In short, the modern study demonstrated a largely positive impact of the COVID-19 pandemic on online medical education.

## Introduction

A growing number of colleges and universities have been implementing a transition from traditional face-to-face teaching methods to online teaching or a combination of online and traditional teaching (blending) [1]. The blended method of teaching involves replacing part of the face-to-face interaction with online instruction [2]. As online education continues to grow, a study in the United States has reported that many educators are just beginning to transform their face-to-face teaching to an online environment [3].

Currently, the world is responding to a pandemic of contagious respiratory disease caused by a novel coronavirus, named COVID-19 [4]. On March 11, 2020, the World Health Organization declared the coronavirus outbreak a pandemic, i.e., the worldwide spread of a new disease [5,6]. The subsequent implementation of social distancing (i.e., increasing the physical space between people) during the COVID-19 pandemic has forced colleges and universities to empty their classrooms and keep the students away from the institutions [7]. Consequently, there has been a general shift from traditional face-to-face instruction to online teaching [8]. Most institutions, including Alfaisal University, have switched to distance learning in the simplest and most convenient ways possible, including conferencing platforms, email, and phone [8].

There have been two main reported predictions about the potential impact of COVID-19 pandemic on online education. Some experts have predicted that the COVID-19 pandemic would adversely impact online education for several reasons [7]. Firstly, they felt that the transition to online education can be challenging, even when the transitioning process is given enough time [9]. Besides, during the COVID-19 pandemic, there is not a single part of the economy that has been unaffected. As a result, college students have become financially vulnerable [10]. Some of the students are worried that they will no longer be able to afford college after the pandemic. Also, being confined at home, some of the faculty and students have been busy trying to manage their children, other elders, or siblings in the house who are also not in school [10].

Challenges to online education reported in the medical literature so far include issues relating to time management, use of technology tools, students’ assessment, communication, and the lack of in-person interaction [9,11]. Besides, online education may not be equitable in terms of access and the quality of teaching [10]. Some students do not have access to laptops, or high-speed internet at home. Also, older internet users benefit the least from online education for reasons such as technophobia [12]. Many teachers themselves are technophobic, i.e., they are worried or not confident enough about dealing with computer hardware and software in their classrooms [13]. Challenges to the online environment during an emergency may delay the adoption of technology-enabled education [14].

In contrast, other commentators have predicted that the COVID-19 pandemic would have a positive impact that will lead to wider acceptance of online and technology-enabled education [8]. Even before COVID-19, there was already a high growth and adoption of education technology [1,3]. Advocates of this viewpoint believe that online education is as effective as traditional classroom education [15]. Moreover, switching over to online instruction during an emergency acts as a reset button to the ailing traditional educational system [10]. The transition to online education requires the faculty’s support in the universities planning such a transition. Of note, 80% of institutions surveyed in a study conducted in the United States confirmed that faculty members are being offered some support for their online courses [16]. The present study aimed to determine and analyze the impact of the COVID-19 pandemic on online education at the College of Medicine (COM) of Alfaisal University in Riyadh, Saudi Arabia.

## Materials and methods

This study was sponsored by Alfaisal University and conducted with the approval of the university’s Institutional Review Board (IRB-20027). This was a cross-sectional study and was conducted from March through May 2020. Located in Riyadh, Saudi Arabia, Alfaisal University is a private, nonprofit, research-based university with five affiliated colleges and over 3,000 students from over 40 nations [17]. The COM offers Bachelor of Medicine and Bachelor of Surgery (MBBS) program, and several master’s degree programs, including a Master of Public Health (MPH) degree program [17].

The study investigators developed a self-administered online questionnaire using Google Forms® (Google LLC, Mountain View, CA). The survey contained an introductory paragraph that informed participants of the study’s aims, the confidentiality of their responses, and the freedom to decline to answer any question or to withdraw from the study altogether. The questionnaire comprised a combination of closed- and open-ended questions. The closed-ended questions included inquiries into participants’ demographics and their experiences before and during the COVID-19 pandemic. Open-ended questions were designed to elicit descriptive feedback in the participants’ own words instead of simple “yes” or “no” answers. As a part of the questionnaire validation process, we invited four faculty members and two students to pilot-test the initial survey draft. The survey was modified based on their feedback.

To launch the survey, we sent an introductory email to members of the target population, which consisted of faculty and students of the COM. The initial email informed the target population of the study’s aims and solicited their participation. Next, the survey’s web link was sent to the target population using the institutional email system. Subsequently, two follow-up email reminders were sent to the same groups. We excluded from participation faculty and students who had participated in the pilot-test of the study questionnaire.

The present study targeted students and faculty who belonged to different generations. The generations were classified as follows based on the year of their birth: Baby Boomers (1946-1964), Generation X (1965-1980), the Millennials or Generation Y (1981-1996), and Generation Z or Post-Millennials (1997-2012) [18]. Baseline and outcome characteristics were summarized using descriptive statistics, as appropriate.

## Results

A total of 208 individuals responded to the survey: 38 (18.3%) faculty, 31 (14.9%) master’s students, and 139 (66.8%) medical students. The response rates were approximately 53.5%, 62.0%, and 11.9% for faculty, master’s students, and medical students, respectively. Of the respondents, 114 (54.8%) were females, 125 (60.1%) were born between 1997-2012 (Generation Z or Post-Millennials), and 66 (31.7%) were born between 1981-1996 (Generation Y or Millennials). The characteristics of the study respondents are depicted in Table 1.

**Table 1:** Characteristics of the study respondents (N = 208)

Characteristics	N (%)
Gender	
Female	114 (54.8)
Male	94 (45.2)
Year of birth	
1946-1964 (Baby Boomers)	8 (3.9)
1965-1980 (Generation X)	9 (4.3)
1981-1996 (Generation Y or Millennials)	66 (31.7)
1997-2012 (Generation Z or Post-Millennials)	125 (60.1)
Position	
Faculty	38 (18.3)
Master's students	31 (14.9)
Medical students	139 (66.8)

Among the respondents, 130 (62.5%) preferred combining online with traditional face-to-face instruction, 53 (25.5%) preferred traditional face-to-face instruction, and only 25 (12.0%) preferred online instruction alone. The acceptance of blending online and face-to-face instruction has been growing in the academic community [2]. Participants’ teaching/learning preferences before the COVID-19 pandemic are presented in Table 2.

**Table 2:** Teaching/learning preferences before the COVID-19 pandemic COVID-19: coronavirus disease 2019

Preferences	N (%)
Combining online with face-to-face instruction	130 (62.5)
Face-to-face instruction	53 (25.5)
Online instruction	25 (12.0)

**Figure 1:**
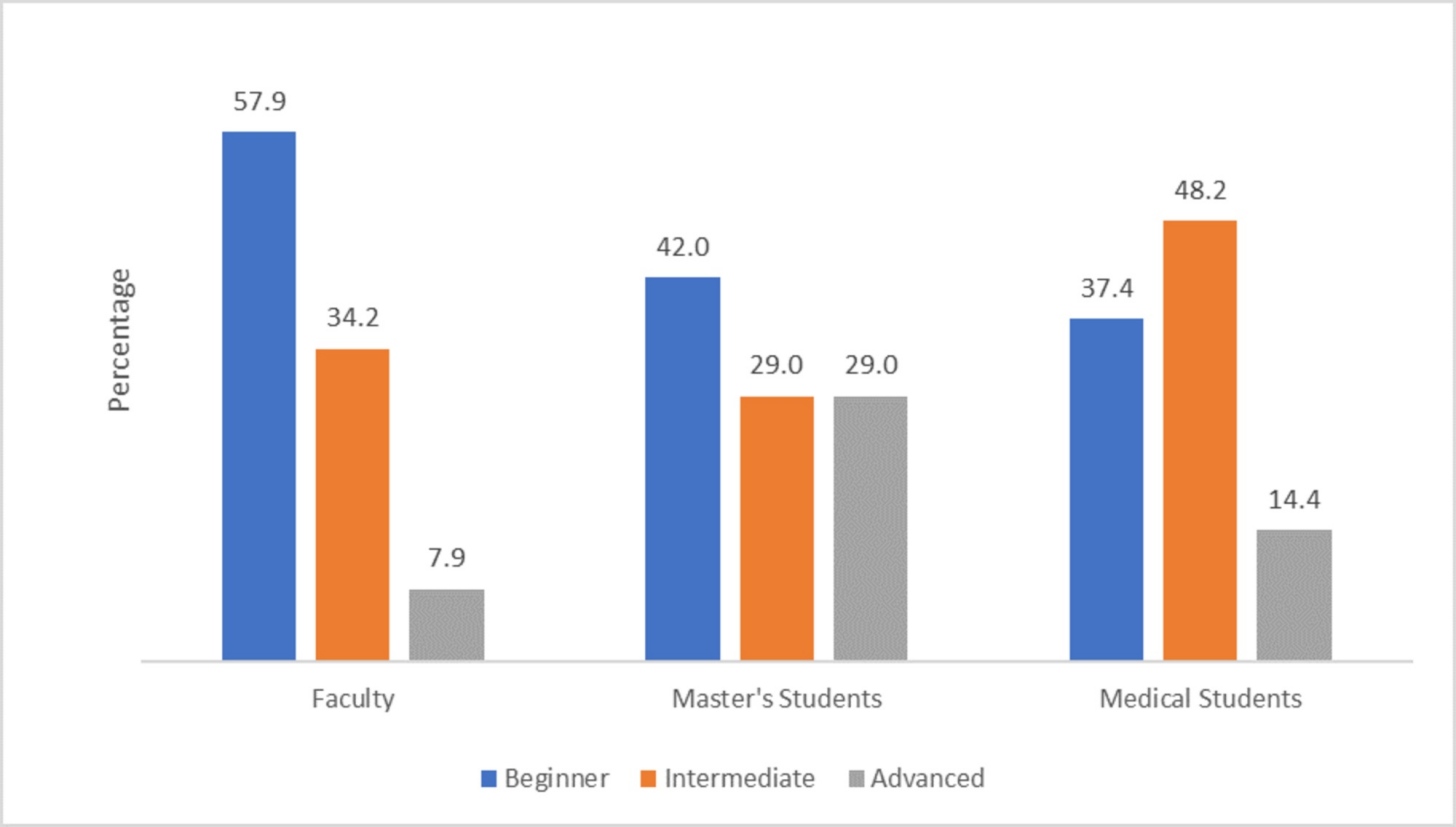
Online learning/teaching experience before the COVID-19 pandemic by position COVID-19: coronavirus disease 2019

When asked to describe their online teaching/learning experience before the pandemic, 57.9% of the faculty, 42% of master’s students, and 37.4% of medical students reported having little or no experience. Online teaching/learning experience before the COVID-19 pandemic by position is presented in Figure 1.

The reported challenges to online education during the COVID-19 pandemic at this institution included issues regarding in-person communication (59%), student assessment (57.5%), use of technology tools (56.5%), experience in online education (55.0%), pandemic-related anxiety and stress (48%), learning curve (35.5%), time management (35.0%), students' evaluations of faculty (24.0%), and technophobia (17.0%). Challenges to online education during the COVID-19 pandemic are presented in Table 3.

**Table 3:** Challenges to online education during the COVID-19 pandemic (N = 200)* *Eight responses were missing; **Check all that apply COVID-19: coronavirus disease 2019

Challenges	N (%)**
Communication	118 (59.0)
Student assessment	115 (57.5)
Use of technology tools (access to hardware and software)	113 (56.5)
Experience in online teaching/learning	110 (55.0)
Mental health (stress, anxiety)	96 (48.0)
Learning curve (adapting to unfamiliar technology)	71 (35.5)
Time management	70 (35.0)
Students' evaluations of faculty	48 (24.0)
Technophobia	34 (17.0)
Other	9 (4.5)

**Figure 2:**
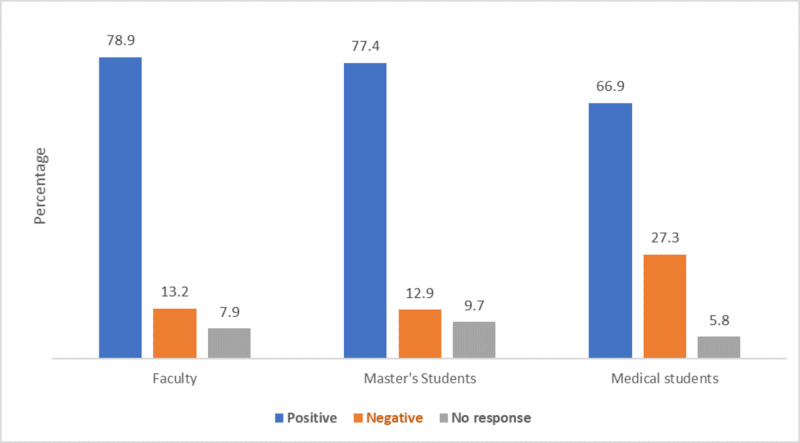
Impact of the shift to online education during the COVID-19 pandemic by position COVID-19: coronavirus disease 2019

The main question in the present study was about assessing the impact of the shift to online education during the pandemic. Remarkably, 70.7% of the study respondents reported increased confidence in the effectiveness of online teaching and learning during the first few weeks of the COVID-19 pandemic. Specifically, 78.9% of faculty, 77.4% of master's students, and 66.9% of medical students reported a positive view of the pandemic's impact. The attitudes toward the impact of the shift to online education during the COVID-19 pandemic by position are presented in Figure 2. Most respondents (76%) intended to integrate online expertise gained during the COVID-19 pandemic into their teaching/learning strategies. The views on willingness to integrate online expertise garnered during the COVID-19 pandemic into practice are presented in Table 4.

**Table 4:** Willingness to integrate online expertise garnered during the COVID-19 pandemic into practice COVID-19: coronavirus disease 2019

Views	N (%)
Agree	158 (76.0)
Disagree	31 (14.9)
No opinion	19 (9.1)

The study survey also solicited participants’ feedback regarding their experience in online education during the COVID-19 pandemic. Most of the comments were in support of either online or blended instruction. Interestingly, most of the faculty were in favor of switching to online teaching. A participant commented that the conditions created by the COVID-19 pandemic proved the effectiveness of online learning. Others reported additional advantages to online learning during the pandemic, including the promotion of self-discipline and responsibility.

Similarly, master’s and medical students gave positive feedback regarding online teaching and even suggested a post-pandemic blended strategy. They believed that online instruction is superior to face-to-face instruction. However, some medical students reported negative experiences with online learning during the COVID-19 pandemic. They believed that online education is not suitable for medical students. Others said that online teaching requires extensive training and cannot be adopted over a short period.

## Discussion

We are currently going through an unprecedented academic crisis. COVID-19 has shaken the foundation of our society. Universities and colleges worldwide have been closed, and online education has suddenly become an academic norm. Under these circumstances, educators and students may find the rapid transition to online instruction distracting and frustrating. Based on previous experiences, experts have predicted that it might take 5-10 years to recover from this pandemic [10]. 

A reasonable number of faculty, master’s students, and medical students responded to our survey during a healthcare crisis. Faculty and students participated in this timely study to ensure that their voices are heard. A recent study has revealed that medical students are interested in being part of the decision-making process relating to issues that may impact their education [11]. Thus, it is prudent to engage faculty and students in the process of revamping education for a challenging time. Nonetheless, the low response rate of the medical students was anticipated since medical students were stressed out trying to adapt to new ways of learning in the middle of a pandemic.

Most of the study respondents belonged to the Millennial or Post-Millennial generations. Both generations are social-media savvy; social media is their primary source of communication [18]. This fact may partly explain the acceptance of online teaching and learning by most study respondents. Moreover, most of the present study respondents preferred combining online with face-to-face instruction. Similarly, previous studies have reported that hybrid learning is becoming more accepted among academic communities because it combines “the best of both worlds” [1]. However, the effectiveness of hybrid learning depends on several factors, mainly adequate faculty training and institutional support [19].

In the present study, the primary challenge to online education at COM as reported by the respondents was related to communication. Faculty and students have quickly learned that clear and concise feedback is essential when switching over to a virtual environment during a healthcare crisis. This opportunity to improve ways of communication between faculty and students during COVID-19 could enhance communication in traditional face-to-face courses as well. Other challenges were related to student’s online assessment, access to computer hardware or software, and other technical barriers, the scarcity of experience with online education, pandemic-related anxiety, and technophobia. These challenges were akin to those faced in the transition to online education during non-emergency situations [11,13,9]. In the present study of highly educated subjects, technophobia was the least reported challenge. Technophobia, the fear of using technological tools like computers, was correlated with users’ education [12]. Identifying these challenges may assist in recognizing online teaching and learning practices that can enhance classes even when we return to conventional face-to-face instruction.

During the current healthcare crisis, when the COM faculty and students are faced with the uncertainties regarding remote instruction, close to half of the respondents stated they felt a level of anxiety about how well the semester would go. They reported reaching out to their campus colleagues, administrators, librarians, and information technology (IT) personnel to assist in the transition to online education. Besides, faculty members also reported the need to re-examine their teaching practices. Study respondents also emphasized the need to draw on research to guide the institution and the academic community in times of healthcare crisis, especially at a research-based institution like the Alfaisal University.

Despite these challenges, the COVID-19 pandemic has had a positive impact on the faculty and students’ acceptance of online education. Specifically, 67% of the medical students reported a positive impact of the pandemic on online learning. In a recent survey of more than 400 college students whose schools had recently switched over to online education due to the COVID-19 pandemic, 60% of students reported being somewhat prepared for the switch [20]. Also, most of the study participants envisioned integrating expertise garnered during the pandemic into their future teaching/learning strategies. However, integrating the skills required for online teaching into practice requires adequate faculty professional development [10].

Most of the faculty feedback supported online teaching; good teaching is good teaching, whether it is face-to-face or in a virtual environment. Some of the faculty were satisfied with online teaching despite using teaching methods they had never tried before. Other faculty commented that the COVID-19 pandemic proved the efficacy of online education. Other comments praised the support from the institution during the pandemic, especially regarding making resources available and real-time support services using multiple channels of communication.

However, the students’ comments varied. Some students praised blended education since it removed some of the traditional teaching barriers that do not work for all students. Schools can tailor the learning experience for each student with access to current technologies and the availability of resources. Students also believed that the quality of instruction has not suffered in the shift to an online environment. Others reported that the first few online sessions were problematic. Faculty and students had to adjust to the online environment. Other students were unhappy with the online learning experience. They wanted to return to conventional face-to-face education right after the pandemic.

The study had several limitations. We conducted the study during the first phase of the COVID-19 pandemic at a private institution where most students are financially secure. Besides, the study did not gather information about the needs of students with disabilities during the transition to online courses. Moreover, we did not record students’ grade point average (GPA), as the GPA might have been a modifier of responses. Finally, there are many online universities, but we typically do not value their degrees as highly as those provided by brick-and-mortar universities. Hence, medical faculty and students tend to support the blended experience to a much greater extent than an entirely online one. Consequently, the study may have limited generalizability [21]. Despite these limitations, we believe the study provides relevant insights into the challenges facing online medical education in a time of healthcare crisis.

## Conclusions

Our study revealed that there has been a positive impact of the COVID-19 pandemic on online medical education at Alfaisal University. Challenges brought about by the pandemic included those related to communication, student assessment, use of technology tools, online experience, pandemic-related anxiety or stress, time management, and technophobia. Despite these challenges, the experience respondents have had during the first few weeks of the pandemic has increased their confidence in the effectiveness of online medical education. While pandemics have historically created challenges, identifying these challenges is the first step in converting them into opportunities. One of the chief opportunities is to engage medical students and faculty in transforming the current pandemic-imposed remote medical education into an evidence-based paradigm. However, the study poses more questions than answers. For example, has the conventional medical education, as we know it, changed forever, and is online learning the future of medical education?
